# Healthcare Utilization Among Hispanic Medicare Enrollees with Complex Chronic Conditions: Puerto Rico and Florida

**DOI:** 10.1093/geroni/igaf122.3341

**Published:** 2025-12-31

**Authors:** Matthew Campos, Maricruz Rivera-Hernandez

**Affiliations:** Brown University School of Public Health, Rhode Island, United States; Brown University School of Public Health, Providence, Rhode Island, United States

## Abstract

Despite higher rates of chronic conditions such as dementia and diabetes, Medicare spending per beneficiary in Puerto Rico (PR) remains significantly lower than on the U.S. mainland (Campos & Rivera-Hernandez, 2024). This study compares the prevalence and demographic profile of Medicare fee-for-service (FFS) beneficiaries (65+) with multiple complex chronic conditions (MCCs) in PR to Hispanic FFS beneficiaries in Florida (FL) and examines whether differences in spending may be driven by variations in service use and access. We analyzed data from the 2022 Medicare Beneficiary Summary File (MBSF), including the Cost and Utilization and 30 CCW Chronic Conditions Segments. Eligible beneficiaries were ≥65, Hispanic, non-dual eligible, and continuously enrolled in Medicare Parts A and B. We stratified by MCCs (2+ complex conditions) and assessed service utilization (dichotomous outcomes (zero vs. any use)) across 17 Medicare spending categories. Demographics were compared using Chi-square and ANOVA tests, and logistic regression models estimated service use differences, adjusting for key covariates. Among FFS beneficiaries, 19.15% (n = 4965/25928) of PR beneficiaries had MCCs, compared to 18.26% (n = 12036/65923) in FL. PR beneficiaries were older and more likely to qualify for Medicare through disability insurance benefits (DIB). Chronic kidney disease was the most common complex condition. PR beneficiaries were significantly less likely than FL beneficiaries to have a claim in 16 of 17 spending categories for those with MCCs and in 14 of 17 categories for those without MCCs (both p < .0001). These findings highlight potential access and quality-of-care issues in PR, with implications for Medicare Advantage benchmarks.

